# Identification of avian encephalomyelitis virus in outbreaks among broilers in Morocco between 2020 and 2024

**DOI:** 10.3389/fvets.2026.1870787

**Published:** 2026-09-17

**Authors:** Ahmed Achhal Elkadmiri, Noura Ait Laydi, Faouzi Kichou, Mohamed Mouahid, Mvibudulu Nzuzi, George A. Abernathy, Mariette F. Ducatez, Siham Fellahi

**Affiliations:** 1 Department of Pathology and Veterinary Public Health, Agronomy and Veterinary Institute Hassan II, Rabat, Morocco; 2 Clinique vétértinaire Tit Mellil, Casablanca, Morocco; 3 Cabinet Mouahid, Temara, Morocco; 4 ENVT, INRAE, IHAP, Université de Toulouse, Toulouse, France

**Keywords:** AEV, chickens, encephalomyelitis, histopathology, Morocco, RT-PCR

## Abstract

**Introduction:**

Avian encephalomyelitis (AE) is a viral disease of poultry caused by avian encephalomyelitis virus (AEV; family *Picornaviridae*) that affects multiple avian species and continues to pose economic concerns for the poultry industry. Clinical expression differs with age, with neurological manifestations predominating in young birds, while adult layers mainly exhibit a transient reduction in egg production.

**Methods:**

This study describes the clinical, pathological, and molecular findings associated with AE outbreaks in six Moroccan broiler flocks aged 6–17 days. Histopathological examination was performed on brain tissues, while AEV was detected by RT-PCR and confirmed by Sanger sequencing. Phylogenetic analysis based on partial 5′-UTR sequences was conducted to determine the genetic relationships among the detected isolates.

**Results:**

Affected flocks showed severe neurological signs, including tremors, drowsiness, ataxia, locomotor incoordination, and paresis, leading to reduced feed and water intake. Morbidity reached up to 90%, with cumulative mortality exceeding 25%. Histopathological examination revealed multifocal gliosis, neuronal degeneration and chromatolysis, axonal degeneration, demyelination, and perivascular cuffing, with the cerebellum particularly affected, showing marked Purkinje cell degeneration and glial proliferation. Phylogenetic analysis classified four Moroccan isolates within genotype B, closely related to the Hungarian strain pf-HK1, and two isolates within genotype A, indicating the circulation of two distinct evolutionary lineages in Moroccan broiler flocks.

**Discussion:**

This study provides the first pathological and molecular characterization of avian encephalomyelitis in Moroccan poultry flocks. These findings highlight the need for continued surveillance and further epidemiological investigations, including assessment of breeder immunity and maternal antibody transfer, to improve understanding of AEV circulation and its impact on poultry production in Morocco.

## 1 Introduction

Avian encephalomyelitis (AE) is a highly contagious viral disease of poultry with a worldwide distribution and significant economic impact intensive production systems. The disease affects several bird species in particular: chickens, pheasants, quails, pigeons and turkeys (1, 2). It manifests in young birds as ataxia and rapid tremors, and in adults as a drop in egg production (3).

The causative agent of AE is avian encephalomyelitis virus (AEV), which belongs to the genus *Tremovirus* within the family *Picornaviridae*. The AEV genome consists of a small (~7 kb) positive-sense single-stranded RNA encoding four structural proteins (VP4, VP2, VP3, and VP1) within the P1 region, as well as seven nonstructural proteins derived from the P2 and P3 regions (4). The 5′ untranslated region (UTR) of the AEV genome contains an internal ribosome entry site (IRES), a characteristic shared with other picornaviruses that influences viral translation and pathogenicity (2, 5).

AEV infection may be transmitted through both vertical and horizontal routes. Vertical transmission occurs from infected hens to their progeny via eggs, either on the egg surface (transovum transmission) or within the egg (transovarian transmission) (2). In contrast, horizontal AEV infection occurs through the orofecal route. The virus is excreted in large quantities in the feces, and birds become infected by pecking contaminated litter or by ingesting food and water contaminated with infectious fecal material (2).

Following an incubation period ranging from two to 7 days, birds start to show clinical signs. Regarding vertical transmission, AEV commonly causes mortality during late embryogenesis and may reduce hatchability.

Eggs laid during the period of viremia can produce chicks that are weak or die in ovo. Vertically infected chicks that survive to hatch typically develop classical neuro-logic signs including ataxia, incoordination, head tremors and progressive flaccid paralysis, and severe outbreaks can produce very high morbidity and mortality (morbidity often 40–60%, and mortality in affected chicks averaging ~25% but exceeding 50% in the most severe outbreaks) (6). A small proportion of survivors may develop lens opacities (cataracts) or later blindness. In adult layers, AEV infection usually causes a transient drop in egg production of about 5–10% lasting up to 2 weeks without obvious deterioration of external egg quality; overall hatchability may fall by roughly 4–5% compared with uninfected flocks. Although routine vaccination of breeders markedly reduces clinical disease in progeny, AEV remains widespread and sporadic field outbreaks (including emergence of novel variant strains) continue to be reported worldwide including the emergence of novel variant strains (2, 5, 7, 8).

Recent studies have also begun to elucidate the molecular epidemiology and genomic diversity of AEV. Whole genome analyses have identified new divergent variants circulating in field outbreaks, with notable genetic distances from existing vaccine strains, highlighting the potential for strain variation and implications for disease control (7). Recent phylogenetic studies have documented the genetic diversity of AEV circulating globally, with evidence of multiple genotypes and lineages (8, 9). Some studies have identified amino acid variations in structural proteins in field isolates, and differences in vaccination routes and vaccine strain protection have been reported in the literature (10, 11). These observations underscore the importance of continued molecular surveillance of circulating AEV strains.” Meanwhile, comprehensive genomic surveys in other regions have demonstrated the value of complete genome sequencing in defining the evolutionary relationships and potential evolutionary emergence of novel or variant strains (8). These advances suggest that broader molecular studies are important to understand AEV diversity, evolution, and vaccine efficacy (10).

The present study aimed to investigate and confirm outbreaks of avian encephalomyelitis in broiler flocks in Morocco between 2020 and 2024. This work combined clinical observations, histopathological examination, and molecular detection to identify the presence of avian encephalomyelitis virus (AEV) in affected flocks. In addition, the study sought to document the occurrence of these outbreaks in Moroccan broiler production and provide the first confirmed characterization of AEV infection in this production sector in Morocco.

## 2 Materials and methods

### 2.1 Ethics statement

The samples analyzed in this study were collected during routine diagnostic investigations conducted in commercial broiler flocks, no experimental infections, animal manipulations, or procedures were performed specifically for research purposes. Necropsies, tissue sampling, histopathological examinations, bacteriological analyses, and molecular investigations were carried out as part of standard veterinary diagnostic activities requested by poultry producers and attending veterinarians.

Because this study consisted exclusively of a retrospective analysis of diagnostic material collected prior to the establishment of the national ethical review framework by the Comité d’Éthique pour les Sciences Animales et la Protection des Animaux de Laboratoire (CESASPV) in Morocco in 2023, prospective ethical approval was not required. The study complied with internationally recognized principles for animal welfare and reporting of animal research.

### 2.2 Description of field cases and clinical investigations

Clinical data were collected from six broiler chicken flocks (*Gallus gallus domesticus*) located in different regions of Morocco, including the Northern territories, Berrechid, Casablanca, Tit Mellil, and Beni Mellal, between 2020 and 2024. Data collection was carried out in collaboration with local farmers and practicing veterinarians. This investigation was not designed as an epidemiological study but rather as a descriptive report of field cases.

All affected flocks exhibited neurological manifestations predominantly characterized by ataxia and paralysis, associated with decreased feed and water intake. From each flock, six freshly dead birds (within 1–2 h post-mortem), aged between 6 and 17 days, were collected and submitted to the Avian Pathology Department at Institut Agronomique et Vétérinaire Hassan II, Morocco, for complete necropsy examination.

For molecular investigations, tissue samples were aseptically collected from the central nervous system, including the telencephalon, mesencephalon, metencephalon, and myelencephalon, as well as from selected peripheral organs (proventriculus, duodenum, pancreas, and myocardium). Individual tissue fragments (approximately 1 × 3 cm) were placed in sterile microcentrifuge tubes and immediately stored at −80 °C until further processing.

For bacteriological examination, aseptic swabs from the brain, liver, and heart were inoculated onto 5% sheep blood agar and MacConkey agar and incubated aerobically at 37 °C for 24–48 h. Colony morphology, hemolytic activity, and purity were evaluated, followed by Gram staining and standard biochemical testing where applicable. No significant bacterial growth was detected in the examined samples, and the cultures were considered negative. Pure isolates were recorded and interpreted in correlation with gross and histopathological findings to assess their potential clinical significance.

For histopathological analysis, representative sections of whole organs were fixed in 10% neutral buffered formalin at a tissue to fixative ratio of 1:10 and subsequently processed using routine histological procedures. Following sampling, carcasses were disposed of by incineration in accordance with institutional biosafety and animal health regulations.

To exclude alternative diagnoses, we performed differential diagnostic testing on all sampled birds. Bacteriological culture was negative for all clinically significant bacterial pathogens, ruling out bacterial encephalitis. Histopathological examination excluded lesions consistent with nutritional deficiencies (e.g., vitamin E/selenium deficiency, thiamine deficiency), toxic encephalopathies, or trauma. No evidence of concurrent parasitic infection was detected. These findings, combined with the characteristic histological presentation and positive RT-PCR results, supported AEV as the primary etiological agent.

### 2.3 Histopathology

Tissue samples preserved in 10% neutral buffer formalin were processed according to standard histopathological procedures. Briefly, the tissues were dehydrated through a graded series of ethanol, cleared and embedded in paraffin wax. Serial sections of approximately 5 μm thickness were cut, mounted on glass slides, and stained with hematoxylin and eosin (H&E). Histological examination was performed using light microscopy. Representative micrographs were acquired using a digital camera mounted on the microscope.

Histopathological lesions were semiquantitatively scored to standardize severity assessment across examined tissues. Gliosis, neuronal degeneration, perivascular cuffing, and demyelination were scored on a four-point scale: 0 = absent, 1 = mild (focal, <10% of tissue area), 2 = moderate (multifocal, 10–30% of tissue area), and 3 = severe (diffuse, >30% of tissue area). Lesion distribution across brain regions (telencephalon, mesencephalon, metencephalon, myelencephalon, and cerebellum) was documented for each bird. Peripheral organ lesion severity was also scored using the same system.

### 2.4 AEV detection by RT-PCR and sequencing

Total viral RNA was extracted from 150 μL of each sample using the Nucleo-Spin® RNA Virus kit (Macherey-Nagel, Germany), designed for the isolation of viral RNA from cell-free fluids, according to the manufacturer’s instructions. Detection of avian encephalomyelitis virus (AEV) was performed by one-step reverse transcription polymerase chain reaction (RT-PCR) targeting conserved sequences within the 5′ un-translated region (5′-UTR), using the primer pair AEV-205F and AEV-489R, as previously described by Goto et al. (12).

RT-PCR amplification was carried out using the QIAGEN® OneStep RT-PCR kit in a final reaction volume of 25 μL containing: (i) 1 × OneStep RT-PCR buffer (with 2.5 mM MgCl₂), (ii) 0.4 mM of each dNTP, (iii) 0.4 μM of each forward and reverse primer, (iv) 0.4 μL of the OneStep RT-PCR enzyme mix (Sensiscript® and Omniscript® reverse transcriptases and HotStarTaq® DNA polymerase)and (v) 2 μL of extracted RNA template.

Amplification was performed in a peqSTAR 2X thermal cycler (peqLAB, Germany) using a touchdown PCR protocol as follows: reverse transcription at 50 °C for 30 min; initial denaturation at 95 °C for 15 min; 5 cycles of denaturation at 95 °C for 30 s, annealing at 58 °C for 30 s, and extension at 72 °C for 1 min; followed by 40 cycles of denaturation at 95 °C for 30 s, annealing at 56 °C for 30 s, and extension at 72 °C for 1 min; then 5 cycles with annealing at 54 °C under the same conditions; and a final extension step at 72 °C for 10 min.

PCR amplicons were purified using a NucleoSpin® Gel and PCR Clean-Up kit (Macherey-Nagel, Germany) and subjected to bidirectional Sanger sequencing using the corresponding primers. Sequencing was performed by Eurofins Genomics (GATC Biotech, Germany).

### 2.5 Nucleotide sequences analyses

Raw sequence data were assembled and edited using BioEdit software version 5.0.9. Sequence similarity searches were performed using the Basic Local Alignment Search Tool (BLAST) available through the National Center for Biotechnology Information (NCBI, Bethesda, MD, USA). Nucleotide sequences obtained from RT-PCR amplicons were analyzed in BioEdit (13) and aligned with corresponding reference sequences retrieved from the GenBank database using the ClustalW algorithm.

Phylogenetic analyses were conducted using the Maximum Likelihood method implemented in MEGA version 7.0. The Kimura two-parameter (K2P) substitution model, identified as the best-fit model based on the “Find Best DNA/Protein Models” function in MEGA, was applied. The robustness of the inferred phylogenetic trees was assessed by bootstrap analysis with 1,000 replicates (14).

### 2.6 Sample traceability and data linkage

To ensure transparency and reproducibility, we maintained comprehensive records linking individual birds, tissue samples, organs, molecular results, and phylogenetic assignments. Each sampled bird was uniquely identified by flock number and necropsy number. Tissue samples were aseptically collected from specific organs, labeled, and stored in a dedicated freezer (−80 °C) with complete chain-of-custody documentation. All RT-PCR results were cross-referenced with bird ID and organ source. Phylogenetic sequences generated from multiple organs within the same flock were compared to assess consistency. Complete traceability data, including flock designation, bird identifier, organ sampled, PCR result (positive/negative), and phylogenetic assignment, are provided in Supplementary Table 1.

## 3 Results

### 3.1 Clinical signs and gross lesions

The clinical history of the affected flocks is summarized in Table 1. A total of six flocks exhibited a marked reduction in feed and water consumption, followed by the onset of severe neurological signs (Figure 1). Affected birds displayed tremors, somnolence, locomotor incoordination, and an unstable gait, frequently losing balance during movement. As the condition progressed, birds developed pronounced ataxia and paresis, often assuming lateral recumbency with abnormal positioning of the legs.

**Table 1:** Suspicious cases of AEV confirmed by PCR.

FLOFLOCKSF	Flock 1	Flock 2	Flock 3	Flock 4	Flock 5	Flock 6
Age (days)	14	17	10	15	6	*16*
Flock size	14,000	15,000	17,000	11,000	8,000	*19,000*
Area	CASABLANCA	CASABLANCA	CASABLANCA	KHENIFRA	RABAT	CASABLANCA
Morbidity	90%	75%	70%	80%	*70%*	*80%*
Mortality (rearing)	25%	11%	25%	20%	15%	*20%*
Sequencing	AEV + Genotype B	AEV + Genotype B	AEV + Genotype A	AEV + GenotypeB	AEV + Genotype B	*AEV +* *Genotype A*

**Figure 1:**
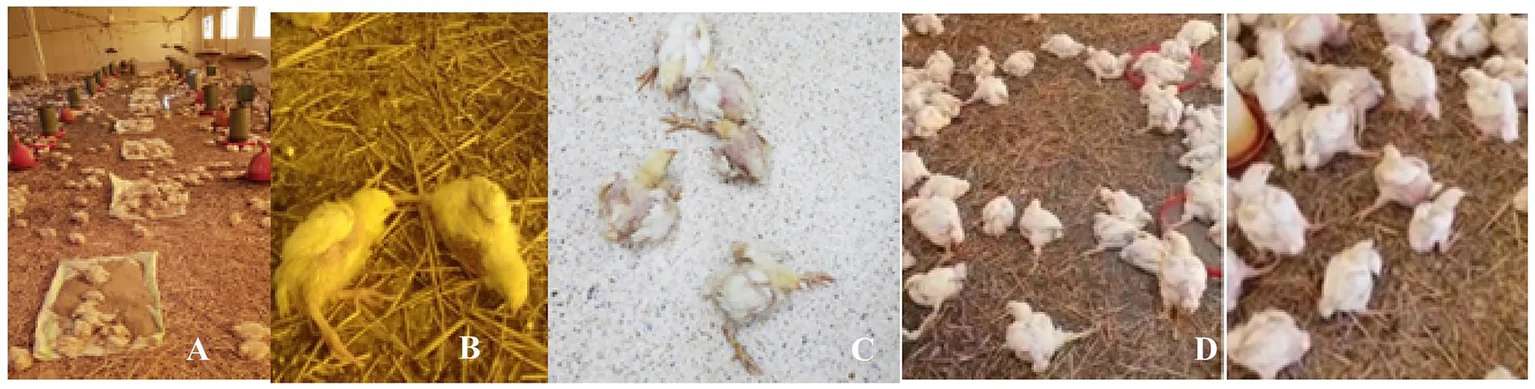
Clinical signs in broiler chicks from flock case 1. **(A)** Broiler barn of flock case 1 showing affected birds and occurrence of high morbidity. **(B)** 11 day old broiler chicks from flock case 1 with ataxia, paresis (lateral recumbency with abnormal positioning of legs). **(C,D)** 14 days old broiler chicks from flock case 1 showing ataxia, paresis (lateral recumbency with abnormal positioning of legs).

In the six affected flocks, birds were between 6 and 17 days of age. Morbidity rates in these flocks reached up to 90%, while cumulative mortality exceeded 25%. Based on the clinical presentation and epidemiological data, avian encephalomyelitis (AE) was strongly suspected, particularly in flocks 1 and 2, as the affected chicks originated from breeder flocks that had not been vaccinated against AEV. Gross pathological examination performed at necropsy revealed no consistent or specific macroscopic lesions in any of the examined organs.

### 3.2 Histopathology

Histopathological examination of the central nervous system revealed lesions that were highly characteristic for avian encephalomyelitis (Figure 2). These changes consisted predominantly of severe, multifocal to diffuse gliosis, accompanied by neuronal degeneration and necrosis (Figure 2). Additional neuropathological features observed in most examined neuronal tissues included central chromatolysis, neuronal satellitosis, axonal degeneration with demyelination, and prominent mononuclear perivascular cuffing (Figures 2A–C).

**Figure 2:**
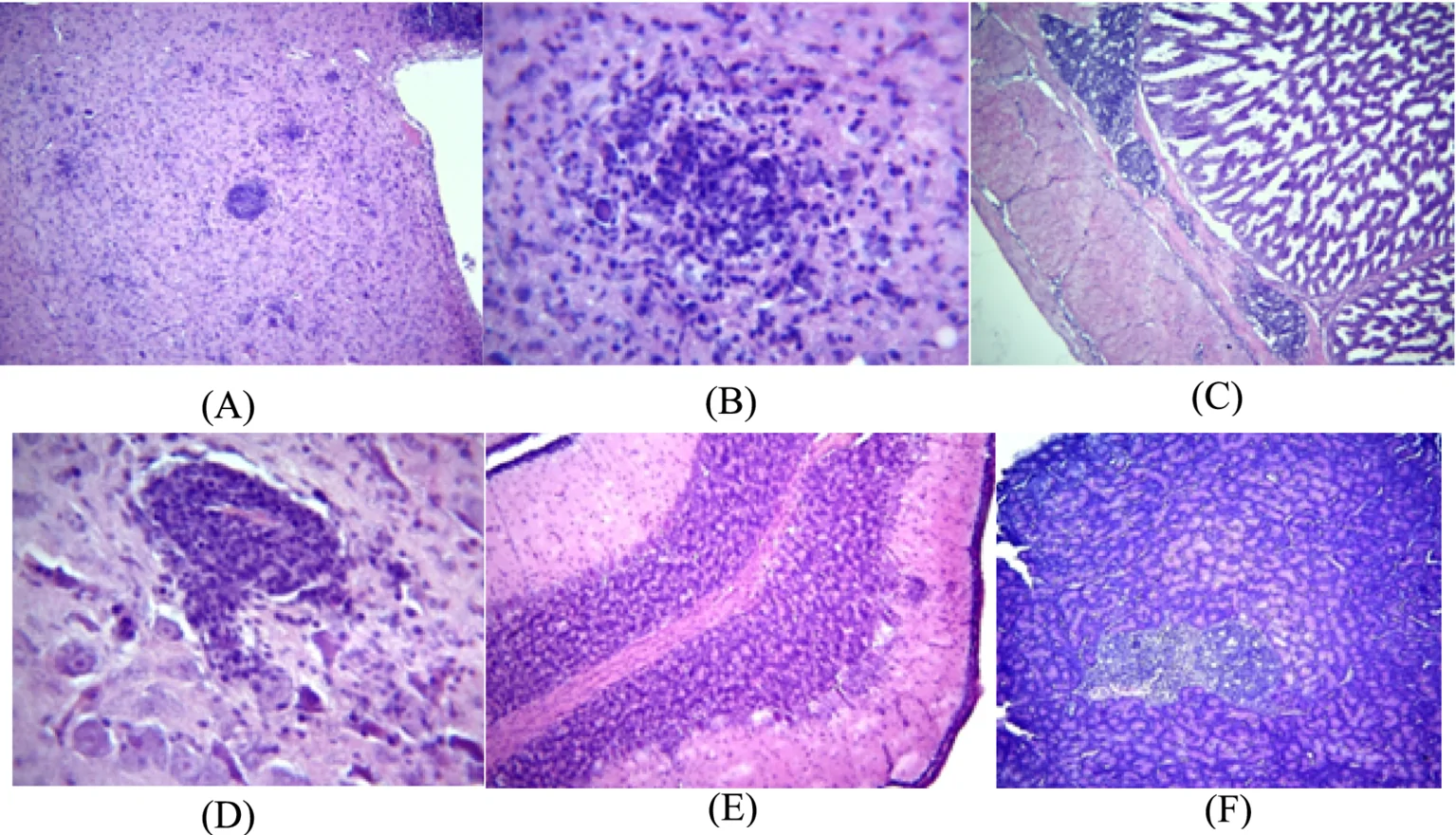
Histopathological examination of central nervous system. **(A)** Brain stem/cerebellum (14 days old broiler chick)—Lymphocytic perivascular cuffing and multifocal and/or diffuse gliosis × 40. **(B)** Cerebellum (14 days old broiler chick)—Multiple clusters of glial cells arising in cerebellar molecular layer and neuronal central chromatolysis × 100. **(D)** Cerebellum (14 days old broiler chick)—Multiple clusters of glial cells arising in cerebellar molecular layer and appearing as flame-like structures, projecting from the granular layer and neuronal central chromatolysis × 100. **(C,E)** Proventriculus (14 days old broiler chick)—Multiple foci of heavy lymphoplasmacytic infiltrates of inner muscularis (C × 40 and E × 100). **(F)** Pancreas (14 days old broiler chick)—Multiple foci of heavy lymphoplasmacytic infiltrates within interstitial tissue × 100.

In the cerebellum, multifocal aggregates of glial cells were observed within the molecular layer, forming characteristic “flame-like” structures extending from the granular layer. These lesions were associated with marked central chromatolysis and degeneration of Purkinje cells (Figure 2D). Encephalitic changes were most pronounced in the myelencephalon, supporting a morphological diagnosis of subacute non-suppurative encephalitis.

Intraneuronal lesions included moderate to severe lymphoplasmacytic infiltrates within the tunica muscularis of the proventriculus (Figure 2E) and the interstitial tis-sue of the pancreas (Figure 2F). Mild inflammatory infiltrates were also detected in the tunica muscularis of the duodenum and in the myocardium. The severity and distribution of encephalitic lesions varied among affected birds within the two examined flocks. No significant microscopic lesions were observed in the remaining organs.

### 3.3 Detection and genetic characterization of Moroccan AEV

Avian encephalomyelitis virus (AEV) nucleic acid was detected by RT-PCR in tissues collected from affected birds. Amplification yielded a specific product of approximately 285 bp, corresponding to the targeted region within the 5′ untranslated region (5′-UTR) of the AEV genome.

Six Moroccan isolates were sequenced: one representative bird from each of the six affected flocks.

The resulting sequences were designated as follows: EHV1.1 (Flock 1), EHV2.1 (Flock 2), EHV3.1 (Flock 3), EHV4.1 (Flock 4), EHV5.1 (Flock 5), and EHV6.1 (Flock 6). Complete sample traceability for all isolates is provided in Supplementary Table 2.

AEV RNA was detected in multiple organs from each bird, including brain, proventriculus, duodenum, and pancreas. Importantly, nucleotide sequences obtained from the five different organs within the same bird and flock were 100% identical within each phylogenetic group, confirming sequence consistency and validating the molecular results.

Phylogenetic analysis based on partial 5′-UTR nucleotide sequences (285 bp amplicon) revealed two distinct evolutionary lineages among the Moroccan isolates: Phylogenetic Group B (Genotype B): Four Moroccan isolates (EHV1.1, EHV2.1, EHV4.1, EHV5.1; corresponding to Flocks 1, 2, 4, and 5) clustered together with the Hungarian reference strain pf-HK1 and other established Group B reference sequences. These isolates showed the highest nucleotide homology with pf-HK1 among the reference strains analyzed (see Supplementary Table 2 for percentage identity values).

Phylogenetic Group A (Genotype A): Two Moroccan isolates (EHV3.1, EHV6.1; corresponding to Flocks 3 and 6) clustered separately within Group A, showing phylogenetic relationships distinct from the Group B isolates and the Hungarian reference strain. These sequences aligned with established Group A reference sequences from GenBank.

The phylogenetic tree with bootstrap support values and explicit group assignments is shown in Figure 3.

**Figure 3:**
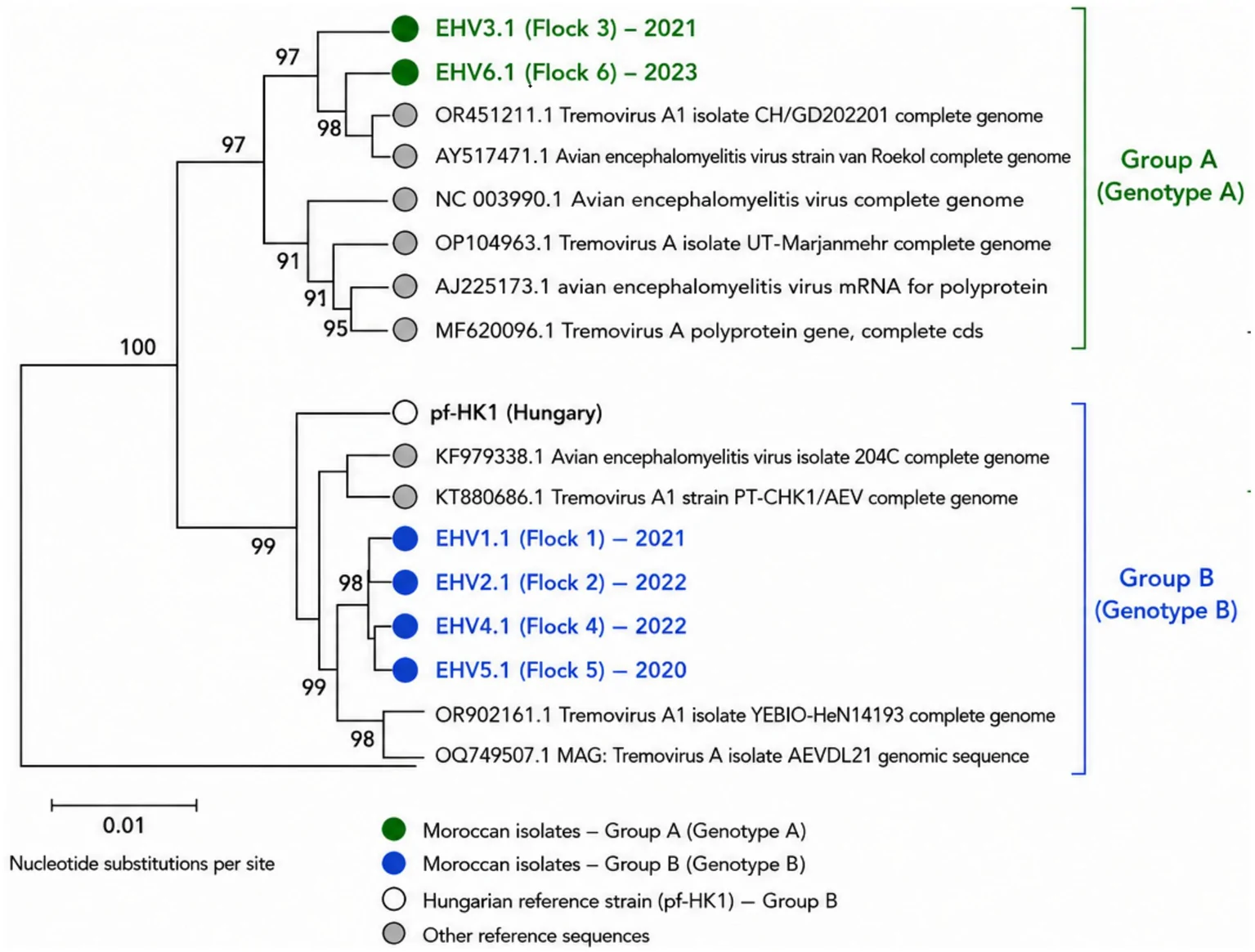
Maximum Likelihood phylogenetic tree of partial 5′-UTR sequences of Moroccan AEV isolates. The tree demonstrates two distinct phylogenetic lineages among the Moroccan isolates. Group B (Genotype B): Four Moroccan isolates [EHV1.1 (Flock 1), EHV2.1 (Flock 2), EHV4.1. (Flock 4), EHV5.1 (Flock 5); shown as blue circles] clustered together with the Hungarian reference strain pf-HK1 (open circle) and other Group B reference sequences (gray). Group A (Genotype A): Two Moroccan isolates [EHV3.1 (Flock 3), EHV6.1 (Flock 6); shown as green circles] clustered separately with established Group A reference sequences (gray). Numbers at nodes indicate bootstrap support values from 1,000 replicates (only values ≥70% shown). Scale bar indicates nucleotide substitutions per site. Phylogenetic analysis was based on 285 bp of the 5′-UTR region using the Kimura two-parameter substitution model as implemented in MEGA7.

## 4 Discussion

Avian encephalomyelitis (AE) remains an infectious viral disease of poultry with considerable economic and sanitary consequences worldwide, particularly in intensive production systems, where early chick mortality and performance losses can be substantial (2, 3, 6). Clinically, AE is classically characterized by neurological manifestations such as ataxia, incoordination, and fine head tremors in young chicks, which has led to its historical designation as “epidemic tremor” (1–3, 6). Despite the longstanding availability of effective vaccines, sporadic outbreaks continue to be reported, highlighting the persistent epidemiological relevance of avian encephalomyelitis virus.

In the present study, we report typical cases of AE in Moroccan broiler chickens, confirmed using an integrated diagnostic approach combining clinical observations, histology, RT-PCR detection, and molecular sequencing. Affected flocks were composed of chicks younger than 3 weeks of age and presented homogeneous neurological signs, including head tremors, drowsiness, ataxia, locomotor incoordination, and progression to paresis and death. These findings are consistent with classical descriptions of AE outbreaks reported in broilers and layers worldwide (6, 15, 16).

The absence of gross pathological lesions, combined with the presence of severe encephalitis characterized by gliosis, neuronal degeneration, central chromatolysis, satellitosis, and perivascular cuffing, further supports AEV as the primary etiological agent. The characteristic “flame-like” glial formations observed in the cerebellum and marked involvement of the myelencephalon are considered pathognomonic for AE and are well documented in both experimental and field cases (2, 3, 11).

AEV can be transmitted both horizontally and vertically, with vertical transmission being an important source of infection in young chicks (2, 17). In the present study, the early appearance of clinical signs, the high morbidity observed, and the epidemiological background of the affected flocks are consistent with a possible vertical transmission of the virus. Although breeder flock vaccination history may be a relevant factor, the available data do not allow us to determine its exact role in these outbreaks. Similar observations have been reported in previous studies (3, 9). Therefore, vertical transmission remains a plausible explanation for the outbreaks described in this study, although further investigations would be needed to confirm this hypothesis. Vaccination of breeder hens before the onset of lay is widely recognized as the cornerstone of AE control, as it prevents vertical transmission by ensuring high levels of maternally derived antibodies in chicks (2). Chicks infected after three to 5 weeks of age typically remain subclinical due to a rapid and effective humoral immune response that limits viral spread to the central nervous system (3). However, accumulating evidence indicates that vaccination does not always confer complete protection under field conditions.

Outbreaks of avian encephalomyelitis have occasionally been reported in progeny originating from vaccinated breeder flocks (15, 17). In this context, Li et al. (10) isolated a field AEV strain (AEV/JS202201) from a vaccinated breeder flock and identified several amino acid substitutions in structural proteins, including a T24A substitution in VP4, relative to the vaccine strain. These observations suggest that genetic variation may occur among circulating AEV strains under certain epidemiological conditions. However, the molecular analysis performed in the present study was limited to a partial 5′UTR fragment and did not include sequencing of structural protein genes, evaluation of maternal antibody transfer, breeder serology, or direct comparison with vaccine strains. Therefore, our findings do not allow conclusions regarding vaccine efficacy, vaccine-strain mismatch, or vaccine-related failure. Nevertheless, the occurrence of these outbreaks emphasizes the importance of maintaining adequate breeder immunization programs and ensuring effective transfer of maternal antibodies to progeny, both of which are critical for the prevention of early AEV infection.

Beyond viral genetics and vaccination protocols, host and management related factors may modulate disease expression. Abbasabadi BM et al. (18) described a clinical AE outbreak in broilers linked to concurrent vitamin A deficiency in breeder hens, despite appropriate vaccination. This finding underscores the importance of breeder nutritional status in sustaining optimal immune responses and preventing vertical transmission. Similar multifactorial interactions between nutrition, immunity, and viral infection have been proposed in other vertically transmitted poultry diseases (6).

Our molecular analysis based on a partial 5′UTR fragment revealed a high nucleotide identity among Moroccan AEV isolates obtained from different organs and flocks. Phylogenetic analysis showed that the sequenced isolates clustered within two phylogenetic groups (Groups A and B), with the Group B isolates showing the highest nucleotide similarity to the Hungarian isolate pf-HK1. These findings indicate that our AEV strains share genetic relationships with previously reported international strains and provide preliminary information on the molecular diversity of AEV circulating in Morocco (9, 12). However, because the analysis was based on a short 5′UTR fragment, the observed clustering should be interpreted as a preliminary phylogenetic characterization rather than definitive evidence of genotype diversity, evolutionary relationships, or antigenic variation. Further studies based on larger genomic regions or complete genome sequences are required to better understand the genetic diversity and epidemiology of AEV in Morocco.

Taken together, our findings provide the first confirmed pathological and molecular characterization of avian encephalomyelitis outbreaks in Moroccan broiler chickens. The results suggest a possible vertical origin of infection and emphasize the need for continued surveillance, monitoring of breeder immunity, and evaluation of maternal antibody transfer. Further molecular and epidemiological investigations are warranted to improve our understanding of AEV circulation in Moroccan poultry production systems.

This study is characterized as a descriptive report of field cases rather than a comprehensive epidemiological investigation. Molecular analysis was limited to partial 5′-UTR sequencing; complete genome sequencing would enable more robust phylogenetic inference and identification of virulence-associated mutations. Vaccine strain compatibility assessment was not performed and remains an important area for future investigation. These limitations should be considered when interpreting the significance of the molecular findings and their implications for AE prevention strategies in Morocco.

## 5 Conclusion

The detection of avian encephalomyelitis virus genotypes A and B in Moroccan broiler flocks provides new insights into the molecular epidemiology of the disease and supports the need for ongoing genomic surveillance and improved preventive measures.

## Data Availability

The raw data supporting the conclusions of this article will be made available by the authors, without undue reservation.
