# Supplementary material for: Identification of avian encephalomyelitis virus in outbreaks among broilers in Morocco between 2020 and 2024
Source: Front Vet Sci. 2026 Sep 17;13:1870787. doi: 10.3389/fvets.2026.1870787 (PMC13626902; doi:10.3389/fvets.2026.1870787)
Supplement: Supplementary file 1 [file Table_1.docx]

**Supplementary Table 1:** Sample Traceability and RT-PCR Results for Avian Encephalomyelitis Virus Detection

Total birds sampled: 36 (6 birds per flock)

Total tissue samples collected: 180 (5 organs per bird: brain, proventriculus, duodenum, pancreas, myocardium)

All samples tested positive for AEV by RT-PCR: 180/180 (100%)

Birds sequenced: 6 (one representative bird per flock)

Sequences generated: 6 (EHV1.1, EHV2.1, EHV3.1, EHV4.1, EHV5.1, EHV6.1)

Sequence consistency: Sequences from all five organs within the same bird were 100% identical

Genotype distribution: Genotype B (Flocks 1, 2, 4, 5); Genotype A (Flocks 3, 6)

RT-PCR, reverse-transcription polymerase chain reaction; AEV, avian encephalomyelitis virus; ND, not determined; PCR+, tested positive by RT-PCR

| Flock | Age (days) | Bird ID | RT-PCR | Sequence ID | Genotype | Status |
| --- | --- | --- | --- | --- | --- | --- |
| 1 | 14 | 1.1 | Positive | EHV1.1 | B | Sequenced |
| 1 |  | 1.2 | Positive | EHV1.2 |  | Sequenced |
| 1 |  | 1.3 | Positive | EHV1.3 |  | Sequenced |
| 1 |  | 1.4 | Positive | ND |  | PCR+ |
| 1 |  | 1.5 | Positive | ND |  | PCR+ |
| 1 |  | 1.6 | Positive | ND |  | PCR+ |
| 2 | 17 | 2.1 | Positive | EHV2.1 | B | Sequenced |
| 2 |  | 2.2 | Positive | EHV2.2 |  | Sequenced |
| 2 |  | 2.3 | Positive | EHV2.3 |  | Sequenced |
| 2 |  | 2.4 | Positive | ND |  | PCR+ |
| 2 |  | 2.5 | Positive | ND |  | PCR+ |
| 2 |  | 2.6 | Positive | ND |  | PCR+ |
| 3 | 10 | 3.1 | Positive | EHV3.1 | A | Sequenced |
| 3 |  | 3.2 | Positive | ND |  | PCR+ |
| 3 |  | 3.3 | Positive | ND |  | PCR+ |
| 3 |  | 3.4 | Positive | ND |  | PCR+ |
| 3 |  | 3.5 | Positive | ND |  | PCR+ |
| 3 |  | 3.6 | Positive | ND |  | PCR+ |
| 4 | 15 | 4.1 | Positive | EHV4.1 | B | Sequenced |
| 4 |  | 4.2 | Positive | ND |  | PCR+ |
| 4 |  | 4.3 | Positive | ND |  | PCR+ |
| 4 |  | 4.4 | Positive | ND |  | PCR+ |
| 4 |  | 4.5 | Positive | ND |  | PCR+ |
| 4 |  | 4.6 | Positive | ND |  | PCR+ |
| 5 | 6 | 5.1 | Positive | EHV5.1 | B | Sequenced |
| 5 |  | 5.2 | Positive | ND |  | PCR+ |
| 5 |  | 5.3 | Positive | ND |  | PCR+ |
| 5 |  | 5.4 | Positive | ND |  | PCR+ |
| 5 |  | 5.5 | Positive | ND |  | PCR+ |
| 5 |  | 5.6 | Positive | ND |  | PCR+ |
| 6 | 16 | 6.1 | Positive | EHV6.1 | A | Sequenced |
| 6 |  | 6.2 | Positive | ND |  | PCR+ |
| 6 |  | 6.3 | Positive | ND |  | PCR+ |
| 6 |  | 6.4 | Positive | ND |  | PCR+ |
| 6 |  | 6.5 | Positive | ND |  | PCR+ |
| 6 |  | 6.6 | Positive | ND |  | PCR+ |
