# Supplementary material for: Identification of avian encephalomyelitis virus in outbreaks among broilers in Morocco between 2020 and 2024
Source: Front Vet Sci. 2026 Sep 17;13:1870787. doi: 10.3389/fvets.2026.1870787 (PMC13626902; doi:10.3389/fvets.2026.1870787)
Supplement: Supplementary file 2 [file Table_2.docx]

**Supplementary Table 2:** Detailed Isolate-by-Isolate Molecular Characterization of Moroccan AEV

| Flock | Year | Location | Organ (Primary) | RT-PCR Ct | Sequence ID | %Identity to pf-HK1 | % Identity to Closest Ref. | Assigned Group | Bootstrap Support |
| --- | --- | --- | --- | --- | --- | --- | --- | --- | --- |
| 1 | 2021 | Casablanca | Brain | 18.2 | EHV1.1 | 94.5 | 94.5 (pf-HK1) | Group B | 98% |
| 2 | 2022 | Casablanca | Brain | 17.5 | EHV2.1 | 94.2 | 94.2 (pf-HK1) | Group B | 99% |
| 3 | 2021 | Casablanca | Brain | 19.2 | EHV3.1 | 78.3 | 98.5 (Group A Ref.) | Group A | 97% |
| 4 | 2022 | Khenifra | Brain | 18.8 | EHV4.1 | 94.1 | 94.1 (pf-HK1) | Group B | 98% |
| 5 | 2020 | Rabat | Brain | 17.3 | EHV5.1 | 94.4 | 94.4 (pf-HK1) | Group B | 99% |
| 6 | 2023 | Casablanca | Brain | 18.4 | EHV6.1 | 76.9 | 97.8 (Group A Ref.) | Group A | 98% |

Ct: Cycle threshold value from RT-PCR

% Identity: nucleotide identity percentage calculated over 285 bp 5′-UTR region

Reference strains: pf-HK1 (Hungarian Group B reference strain); Group A references retrieved from GenBank
